# Molecular chaperones and their denaturing effect on client proteins

**DOI:** 10.1007/s10858-020-00353-7

**Published:** 2020-11-02

**Authors:** Sebastian Hiller

**Affiliations:** Biozentrum, University of Basel, Klingelbergstr. 70, 4056 Basel, Switzerland

**Keywords:** Molecular chaperones, Protein folding, Protein structure, Proteins dynamics, NMR spectroscopy, Chaotropic denaturants, Thermal unfolding, Protein stability

## Abstract

Advanced NMR methods combined with biophysical techniques have recently provided unprecedented insight into structure and dynamics of molecular chaperones and their interaction with client proteins. These studies showed that several molecular chaperones are able to dissolve aggregation-prone polypeptides in aqueous solution. Furthermore, chaperone-bound clients often feature fluid-like backbone dynamics and chaperones have a denaturing effect on clients. Interestingly, these effects that chaperones have on client proteins resemble the effects of known chaotropic substances. Following this analogy, chaotropicity could be a fruitful concept to describe, quantify and rationalize molecular chaperone function. In addition, the observations raise the possibility that at least some molecular chaperones might share functional similarities with chaotropes. We discuss these concepts and outline future research in this direction.

## Atomic resolution studies of chaperone–client systems

Cells in all kingdoms of life possess helper proteins—the chaperones—to perform essential tasks in the genesis of biomacromolecular structure (Ellis 1993; Bukau et al. 2006; Horwich and Fenton 2009; Hartl et al.. 2011; Georgescauld et al. 2014; Schopf et al. 2017). Chaperones can be classified into two groups, based on the broadness of their substrate range, the clientome. “Specialized chaperones” are specific for a single client or a few clients, while “general chaperones” have large clientomes with up to 100s of members (Bose and Chakrabarti 2017). The functionality of general chaperones is typically transitive among homologues, across species and even between different chaperone types, such that their clientomes overlap at least partially. In vitro assays for chaperone activity, such as the prevention of aggregation of model proteins, frequently show that different general chaperones function similarly, including ATP-independent as well as ATP-dependent ones (Stenberg and Fersht 1997; Gray and Fersht 1993; Gray et al. 1993; Entzminger et al. 2012; Huang et al. 2016; Burmann et al. 2020). Overall, these notions suggest that common biophysical principles are shared between general chaperones.

The best characterized general chaperones are arguable the ATP-dependent Hsp90, Hsp60 and Hsp70 from the Heat shock protein (Hsp) family (Schlecht et al. 2011; Barducci and De Los Rios 2015; Sontag et al. 2017; Wruck et al. 2018; Burmann et al. 2020; Sousa et al. 2016; Goloubinoff et al. 2018; Rebeaud et al. 2020; Schopf et al. 2017). These large molecular machines integrate with several cofactors (co-chaperones) towards dedicated functional cycles, often in a modular fashion. Other general chaperones of high biological interest include the ATP-independent prefoldin, trigger factor, SecB, and the bacterial periplasmic chaperones SurA, Spy and Skp. These chaperones feature the “holdase” function—the ability to bind unfolded and partially folded clients for extended time periods.

As for all biomacromolecules, resolving structural and functional features at atomic resolution is key to understanding the biological function. For many of the key chaperones, atomic structures of the client-free forms have long been available (Braig et al. 1994; Xu et al. 2000; Bitto and McKay 2002; Ferbitz et al. 2004; Korndörfer et al. 2004; Walton and Sousa 2004; Webb et al. 2006; Ali et al. 2006; Zhang et al. 2010; Kityk et al. 2012). In contrast, structural descriptions of client-interacting states have long escaped experimental access, arguably so, because chaperone-bound clients are highly dynamic and frequently adopt multi-conformational ensembles, rendering them unsuitable for coherent averaging of diffraction or transmission data (Hiller and Burmann 2018). For example, cryo-electron microscopy could detect client proteins bound to Hsp60 or Hsp90 only at low resolution, preventing determination of atomic coordinates (Clare et al. 2009; Chen et al. 2013; Verba et al. 2016; Cuéllar et al. 2019).

**Fig. 1:**
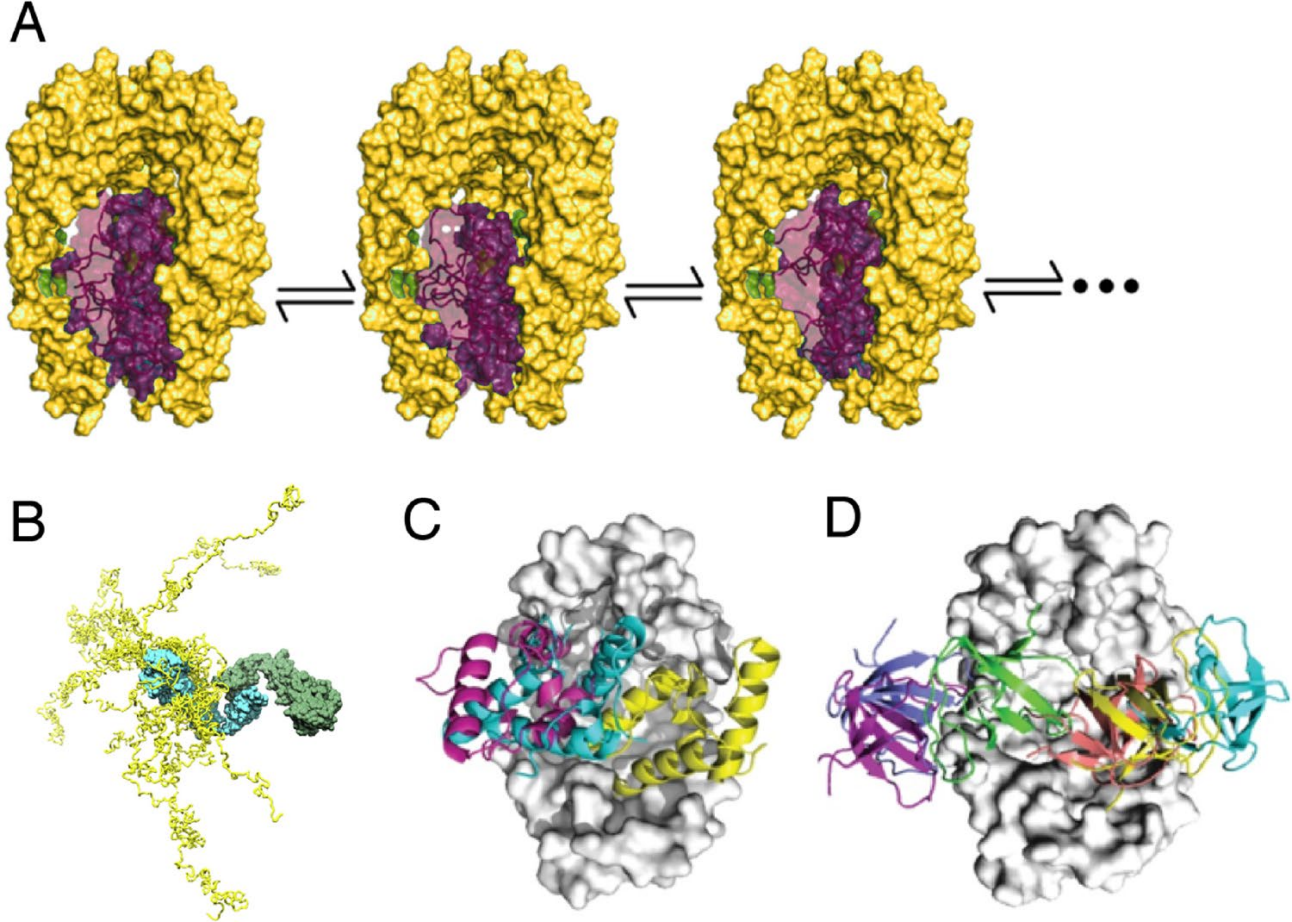
Dynamic ensemble models of general chaperones in complex with clients. **a** Bacterial chaperone Skp (yellow) with the bound client protein OmpX (purple) (Burmann et al. 2013). **b** Conformational ensemble of the intrinsically disordered protein Tau (yellow) bound to chaperone Hsp90 (cyan/green) (Karagöz et al. 2014). **c**, **d** Chaperone Spy (grey) with bound client Im7 (**c**) or SH3 (**d**) (He et al. 2016; He and Hiller 2018, 2019). Different conformations of the bound client are labeled by different colors. See original publications for all details. Reproduced with permissions

The situation changed substantially in the early 2010s with the success of modern high-resolution techniques of NMR spectroscopy. Advanced isotope labelling combined with spectroscopic techniques including TROSY had raised the size limitations for functional and structural studies of biomolecular systems towards the hundreds of kDa, in particular for systems with a known ground structure (Pervushin et al. 1997; Tugarinov et al. 2003; Sprangers et al. 2007; Mas et al. 2013; Goto et al. 1999). When combined with advanced biochemical preparations, these techniques thus enabled the first complete description of a full-length client in complex with a chaperone, the Omp–Skp system (Burmann et al. 2013) (Fig. 1a). In the following years, client-bound states of multiple chaperones were resolved to atomic level, including trigger factor, SecB, Tim9/10, Spy, Hsp40, Hsp70 and Hsp90 (Saio et al. 2014; Karagöz et al. 2014; Huang et al. 2016; Salmon et al. 2016; He et al. 2016; Rosenzweig et al. 2017; Weinhäupl et al. 2018; Jiang et al. 2019) (Fig. 1b–d). In many of these cases, the client proteins bound to the chaperone adopt dynamic ensemble states. Notably, the dynamic interconversion of conformations while remaining bound to the chaperone is in full agreement with a number of functional studies (Gray and Fersht 1993; Gray et al. 1993; Stenberg and Fersht 1997; Burmann et al. 2013; Thoma et al. 2015; He et al. 2016; Stull et al. 2016; Horowitz et al. 2017; Hiller and Burmann 2018), highlighting client fluidity as a central feature for many chaperones and the need to describe them by ensembles rather than static structures. Additional notable NMR studies yielded valuable insights into further functional aspects of general chaperones, including the Hsp60 system (TRiC/CCT (Joachimiak et al. 2014), GroEL (Libich et al. 2015, 2017) and thermosome (Mas et al. 2018)), Hsp70 (Zhuravleva et al. 2012; Libich et al. 2013; Rosenzweig et al. 2013; Sekhar et al. 2015), and Hsp90 (Park et al. 2011; Oroz et al. 2017). Overall, from these high-resolution studies, common features emerged that connect the functionality of several chaperones. We discuss these two of these features in the following.

## Solubilization of aggregation-prone polypeptides

**Fig. 2:**
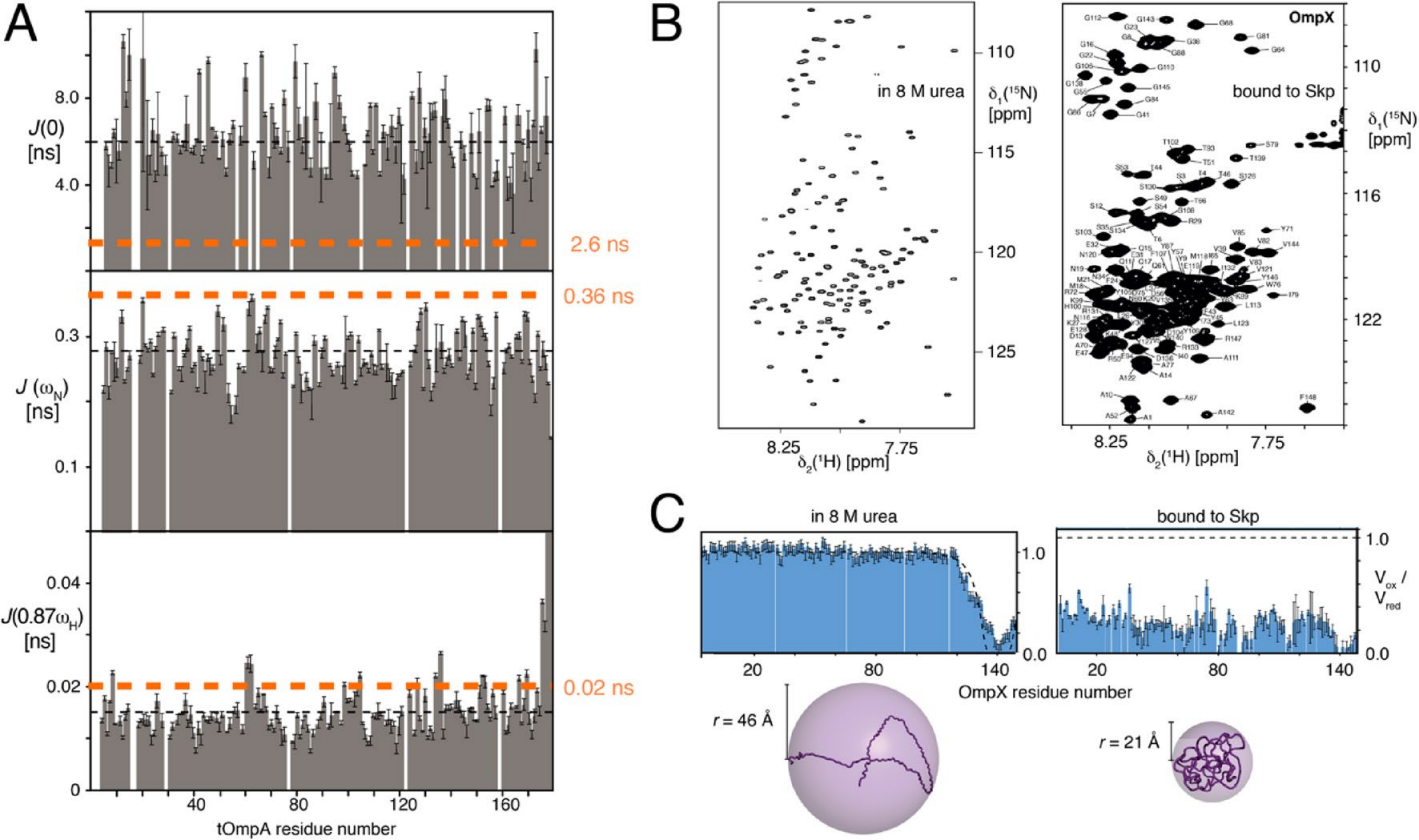
Similarities of chaperone- and urea-solubilized client states. **a** Residue-specific backbone dynamics of tOmpA bound to chaperone Skp. The spectral density function *J*(ω) is evaluated at three timescales. The average values bound to Skp is indicated by a black dashed line and in 8 M urea by an orange dashed line. **b** Similarity of 2D [^15^N,^1^H]-TROSY spectra of OmpX in chaperone and in urea. **c** Compactness of the OmpX polypeptide in 8 M urea solution (left) and bound to chaperone Skp (right) as determined by intramolecular PRE. Low V_ox_/V_red_ values indicate special proximity of the residue to the paramagnetic probe at residue 140. A single model conformation of the polypeptide is shown below for each case and the corresponding radius of gyration *r* is indicated. See original publications for all details. Adapted from (Burmann et al. 2013) and (Tafer et al. 2004) with permissions

The first common feature is the ability to solubilize aggregation-prone polypetides that would otherwise readily precipitate in aqueous solution. Among other, this function is performed by the periplasmic chaperone Skp, that solubilizes unfolded outer membrane proteins (Omps) in chemical equilibrium. In the absence of the chaperone, the Omps immediately precipitate in the same aqueous buffer (Burmann et al. 2013; Schiffrin et al. 2016). Thereby, the NMR spectra of the Omp clients bound to the chaperone feature a narrow chemical shift dispersion for all resonances, both in the backbone and the side chains (Fig. 2b). This indicates random-like sampling of backbone dihedral angles in the Ramachandran space with fast kinetics (Burmann et al. 2013; Callon et al. 2014). The presence of fast interconversion dynamics in the clients was further quantified by NMR measurements (Burmann et al. 2013). The backbone dynamics of the Skp-bound clients OmpX and tOmpA on the ps–ns timescale, as described by the spectral density function *J*(ω_N_) and *J*(0.87ω_H_), resembles in magnitude the values that the same polypeptide has in 8 M urea solution, indicating high local flexibility (Fig. 2a). In contrast, the dynamics *J*(0), which correspond to the effective overall molecular tumbling, are strongly elevated compared to the urea-denatured form, reflecting the coupling of the Omp client to the large molecular weight of the chaperone. Notably, despite the fast local dynamics, the chaperone-bound state is spatially compacted relative to the random coil ensemble in the bulk chaotrope urea (Fig. 2c).

**Fig. 3:**
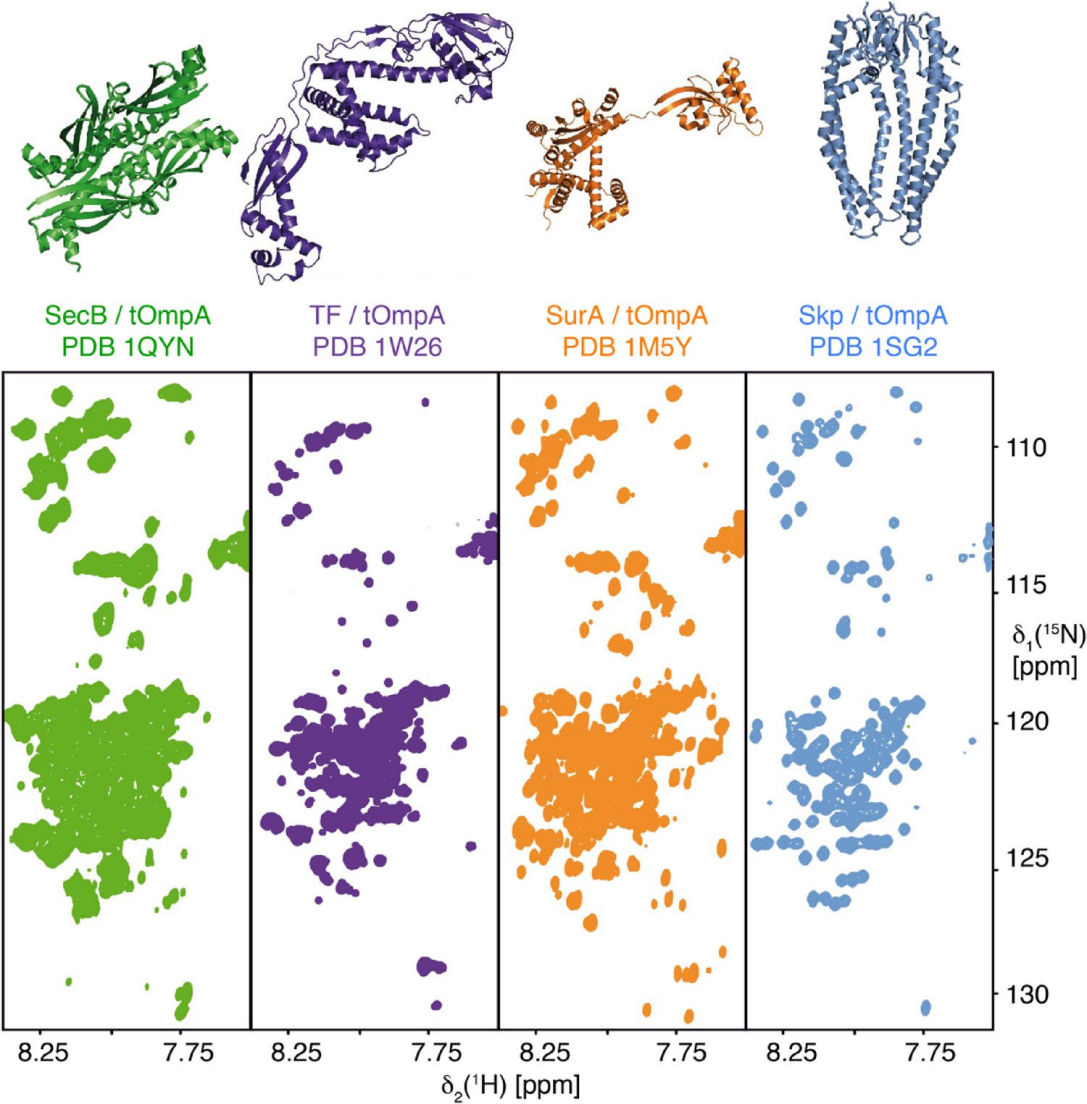
Different chaperones solubilize the membrane protein tOmpA in an unfolded state. 2D [^15^N,^1^H]-TROSY NMR fingerprint spectra of [*U*-^2^H,^15^N]-tOmpA bound to each of the unlabeled chaperones SecB (green), trigger factor (TF, purple), SurA (orange) and Skp (blue). All spectra were recorded at 37 °C in aqueous buffer. The PDB structure of the respective chaperone is shown above each spectrum, indicating their structural diversity. Note that in the absence of chaperone, tOmpA is not soluble in the same buffer. Reproduced from (Burmann et al. 2013) with permission

Compaction of the client and fast local polypeptide dynamics of a chaperone-bound client was also observed for the ATP-independent chaperone Spy interacting with the soluble client protein Im7_M_. Spy compacts the disordered client, but keeps it in an overall dynamic state that reorients on the chaperone surface (He et al. 2016; Stull et al. 2016). For three other molecular chaperones, trigger factor, SurA, and SecB, spectra of the bound outer membrane protein client showed similarly narrow chemical shift dispersion. Just as for the chaperone Skp, these spectra indicate indicating fast averaging of backbone dihedral angles and demonstrate that these chaperones also solubilize the Omp clients for extended time periods in fluidic states (Fig. 3). Taken together, the five chaperones trigger factor, Skp, SurA, SecB and Spy are able to dissolve aggregation-prone polypeptides in aqueous solution in thermal equilibrium.

## Denaturation of folded and partially folded clients

**Fig. 4:**
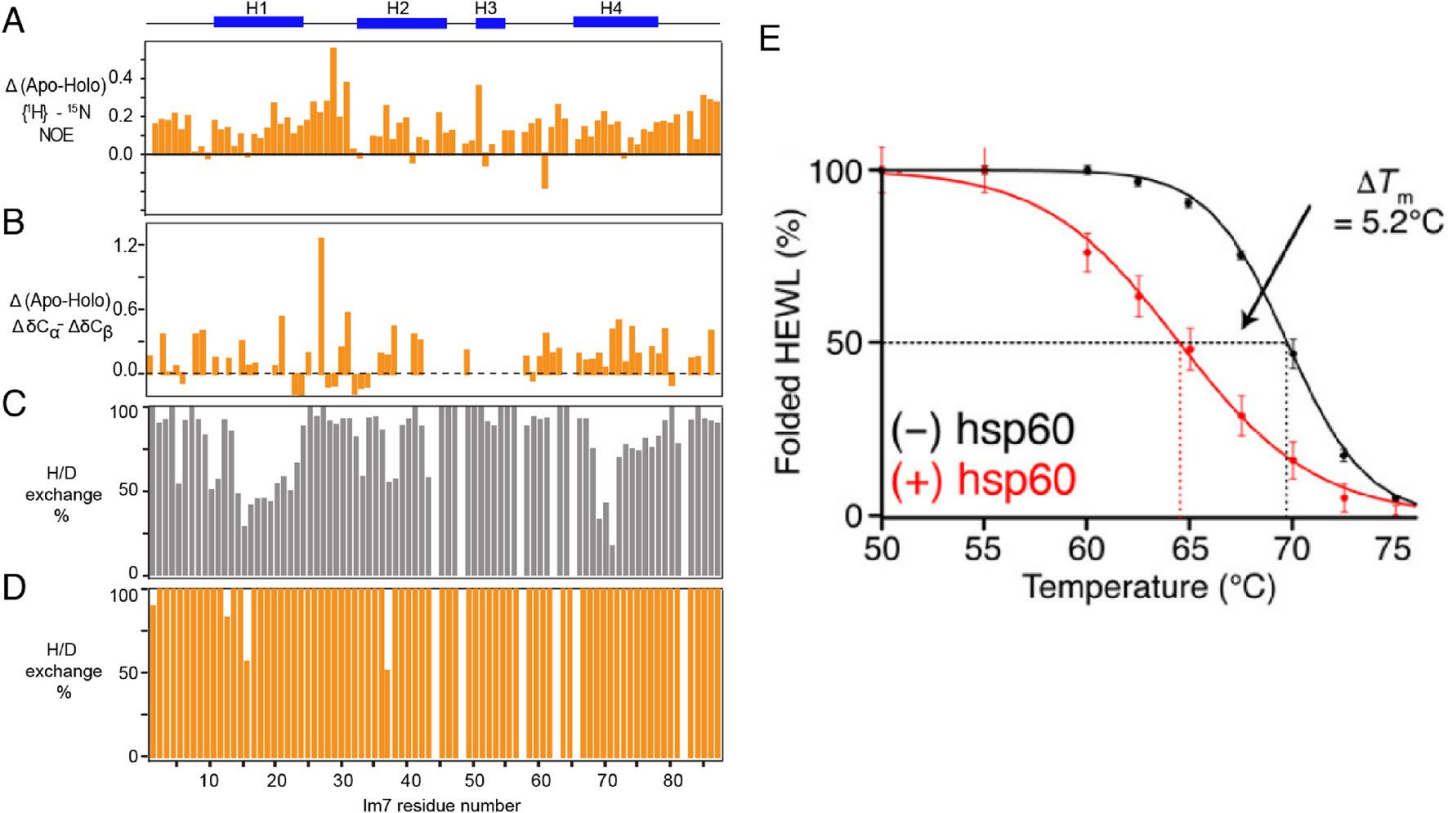
Denaturation of proteins by molecular chaperones. **a**–**d** Effect of the chaperone Spy on the client protein Im7 (He et al. 2016). **a** Difference in heteronuclear NOE of the client upon chaperone binding. A positive value means an increase in local flexibility. **b** Difference in secondary chemical shifts. Positive values indicate a decrease in helical propensity. **c**, **d** Backbone amide proton exchange in absence (**c**, grey) and presence (**d**, orange) of Spy. **e** Effect of the chaperone hsp60 on the melting temperature of the client protein HEWL (Mas et al. 2018). The population of folded and unfolded protein has been quantified as a function of temperature using NMR spectroscopy. See original works for all details. Reproduced from (He et al. 2016) and (Mas et al. 2018) with permissions

A second common functional theme of chaperones that was observed in multiple studies is a denaturing effect on client proteins. In one exemplary work, the effect of the ATP-independent periplasmic chaperone Spy on the partially folded client protein Im7 was characterized by high-resolution NMR spectroscopy (He et al. 2016). Spy was found to interact with Im7 via a specific local region of the client protein and this interaction was shown to induce unfolding of the entire protein. Several spectroscopic parameters evidence this denaturation effect: The local dynamics of the polypeptide on the ps–ns timescale was globally increased, as measured by the heteronuclear NOE (Fig. 4a). Secondary chemical shifts, which are a direct measure for helical secondary structure propensity, were also reduced in the entire protein (Fig. 4b). And thirdly, the kinetic exchange rates of amide protons with water were significantly increased in the entire domain upon interaction with the chaperone (Fig. 4c, d). These data thus indicate that the conformational equilibrium between folded and unfolded Im7 is shifted towards the unfolded conformation by the interaction with the chaperone Spy, corresponding to a denaturation.

The equivalent effect was observed in several independent studies also for very different molecular chaperones, the ATP-dependent Hsp60 family. Early studies using H/D exchange evidenced an unfolding activity of the bacterial Hsp60 variant GroEL on the substrate Rubisco, notably in the absence of ATP or any other source of Gibbs free energy (Shtilerman et al. 1999). The same observation was subsequently also made for other clients, for both GroEL and the Hsp60 form CCT (Priya et al. 2013a, b). More recently, solution NMR spectroscopic observations of the model client protein HEWL (hen egg white lysozyme), showed that archaeal Hsp60 decreased its melting temperature by about five degrees, effectively denaturing the client protein (Mas et al. 2018) (Fig. 3e).

Also for the large chaperone Hsp70, the specific activity to unfold client proteins has been repeatedly reported. When embedded into functional cycles, such unfoldase function of Hsp70 eventually leads to an effective folding reaction for clients, but the elementary underlying function of Hsp70 was shown to unfold rather than to fold the clients (Ben-Zvi et al. 2004; Morán Luengo et al. 2018, 2019). Additional observations of client unfolding activity have also been reported also for Hsp90 and for the small heat-shock proteins (sHsps) (Finka et al. 2016). Overall, these multiple observations evidence a denaturation effect for a majority of the general chaperones, including the most prominent members Hsp60, Hsp70 and Hsp90, as well as multiple classical holdases.

## Chaotropic compounds and their effect on proteins

The terminology of “chaotropicity” was introduced in 1962 to classify compounds that destabilize biomolecular structures in aqueous solution, i.e. decrease their melting temperature (Hamaguchi and Geiduschek 1962). Contemporary lists of chaotropes contain over 60 small molecule substances, including also the Hofmeister series, one of the earliest collections of compounds that modulate the solubility of proteins (Hofmeister 1888; Baldwin 1996; Zhang and Cremer 2009; Cray et al. 2013). In aqueous solutions of chaotropes, the unfolded–folded equilibrium is shifted towards the unfolded state, leading to an effective destabilization of biomacromolecular structure. Notably, for a given chaotrope this effect is not specific to certain targets, but acts in a non-specific fashion on a wide range of biomacromolecules. At the same time, the effect of a chaotrope on a given target protein is non-uniform, as it depends on the presence and the features of local structure. Structured regions are being unfolded, while unstructured regions essentially remain as before. The mechanism of chaotrope action remains puzzling despite intensively being studied (Ball and Hallsworth 2015). Chaotropes have been proposed as perturbers of water structure and thus de-stabilizers of protein structure (Ball and Hallsworth 2015). Thereby, it is not yet clear whether the effects chaotropes have on water are an unavoidable consequence of the effects chaotropes have on biomolecules, or whether the water effects are mechanistically required to then lead to the destabilization of the biomacromolecules and whether all chaotropes necessarily act via the same or similar mechanisms (Ball and Hallsworth 2015). Alternative mechanistic suggestions rely on hydrophobic cavity effects (Breslow and Guo 1990; Baldwin 1996; Graziano 2011), or direct interaction with the polypeptide backbone (Möglich et al. 2005). Molecular dynamics simulations reported that the chaotrope urea destabilizes proteins by both indirect and direct mechanisms, i.e. by perturbation of the water structure and interference with the hydrophobic core of the protein, but also by interactions with the polypeptide backbone (Bennion and Daggett 2003). Irrespective of the underlying mechanism, as a net effect, chaotropes dissolve aggregation-prone segments of polypeptides better than aqueous solution alone, notably without necessarily being hydrophobic substances themselves. The dissolved polypeptides adopt random-coil conformations, emerging from independent local sampling of the Ramachandran space at each polypeptide bond (Shortle 1996; Smith et al. 1996). Furthermore, among the large variety of chaotropes, only a limited subset are able to dissolve aggregation-prone proteins such as membrane proteins in aqueous solution. This subset comprises urea, guanidinium, thioisocyanate and perchlorate. While bulk organic solvents can also dissolve polypeptides in unfolded forms, only the above-mentioned set of chaotropes can do this task in aqueous solution. In other words, the ability to dissolve aggregation-prone peptides constitutes a strong identification criterion for chaotropicity.

## Comparing molecular chaperones and chaotropes

When summarizing the above-mentioned functional properties of chaperones and chaotropes, certain similarities become apparent. Chaotropes and chaperones are the only known molecules that can solubilize aggregation-prone peptides in aqueous solution, and both types of molecules have a denaturing effect on client proteins. These observations thus suggest that chaotropicity might be a fruitful theoretical concept to describe the function of at least some chaperones.

In addition, the similarities between molecular chaperones and classical chaotropes bear the interesting possibility that at least in some cases, certain underlying mechanistic principles might be shared. For ATP-independent chaperones, chaotropicity might well be correlated with the holdase function, in the sense that free energy gained from the chaotrope effect at least partially contributes to the binding affinity between chaperone and client. For more complex chaperones, chaotropicity might be the underlying basic function that is then embedded in regulatory mechanisms to result in complicated functional cycles. Uncontrolled chaotropicity is generally detrimental to cells and the embedding and protection into functional cycles may in many cases serve the purpose to prevent unwanted damaging effects towards off-targets by tight control. Of further note, the concept of chaotropicity as the molecular basis for chaperone function is in full agreement with findings that both hydrophobic and non-hydrophobic interactions are involved in chaperone function (Qu et al. 2007; He et al. 2016; Koldewey et al. 2016).

The concept to consider chaperones chaotropes may bring interesting perspectives for both chaperone and chaotrope research, in particular, since the individual mechanisms underlying the classical chaotropes are also still not fully understood (Ball and Hallsworth 2015). Atomic resolution studies of chaperones and additional biophysical measurements may thus also contribute to better understand the mechanisms underlying classical chaotropes. Because none of the 20 proteinogenic amino acids is itself a chaotrope, evolution appears to have created chaotropicity by suitable spatial arrangement of non-chaotropic elements. We anticipate that combinations of experiments with bioinformatic analyses will eventually reveal the molecular principles for the creation of chaotropic surfaces in chaperones. Along these lines, it should become possible to define a quantitative measure for chaotropicity of chaperones and connect this to classical chaotropes.
